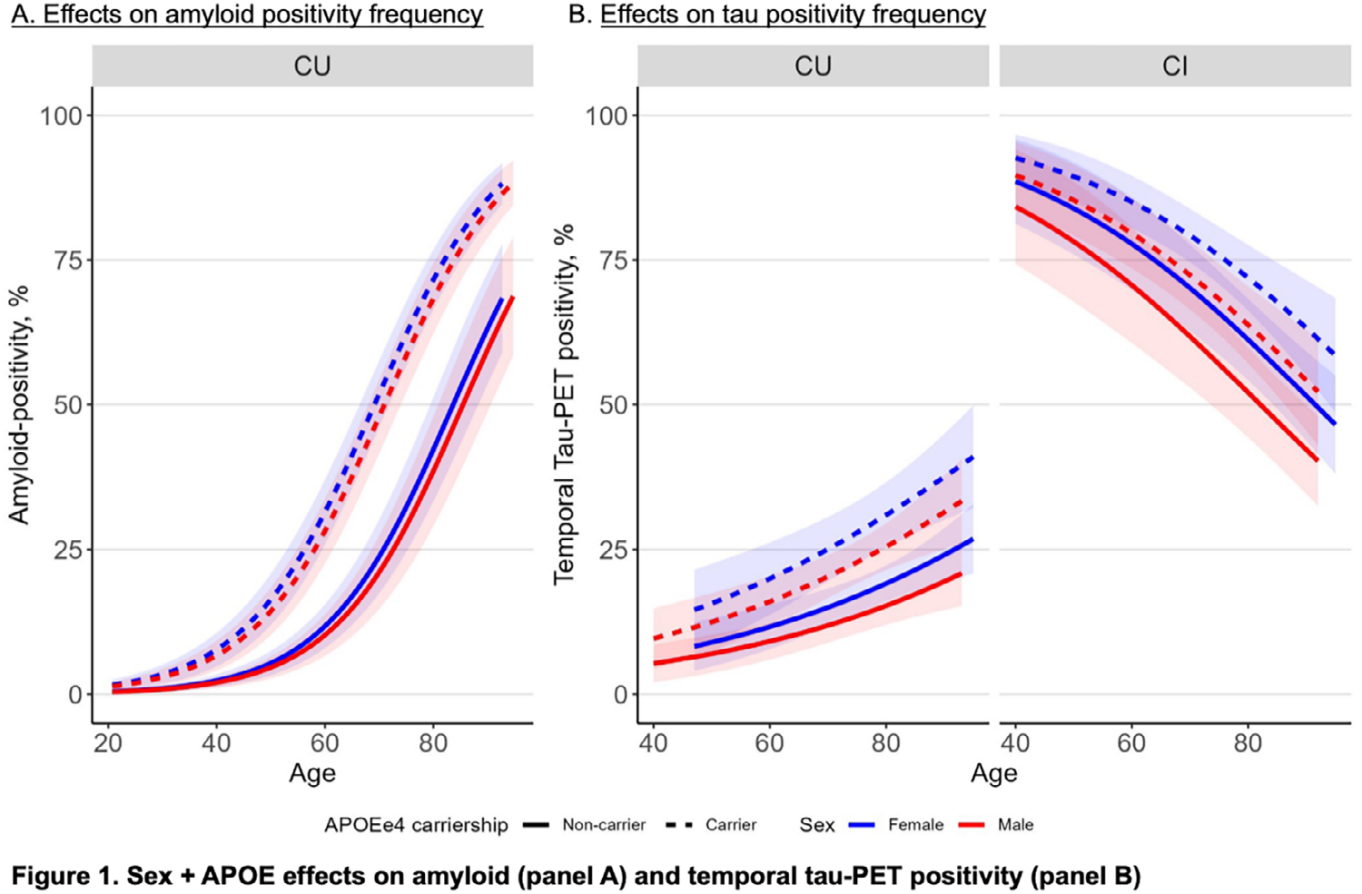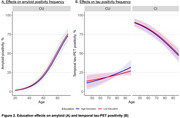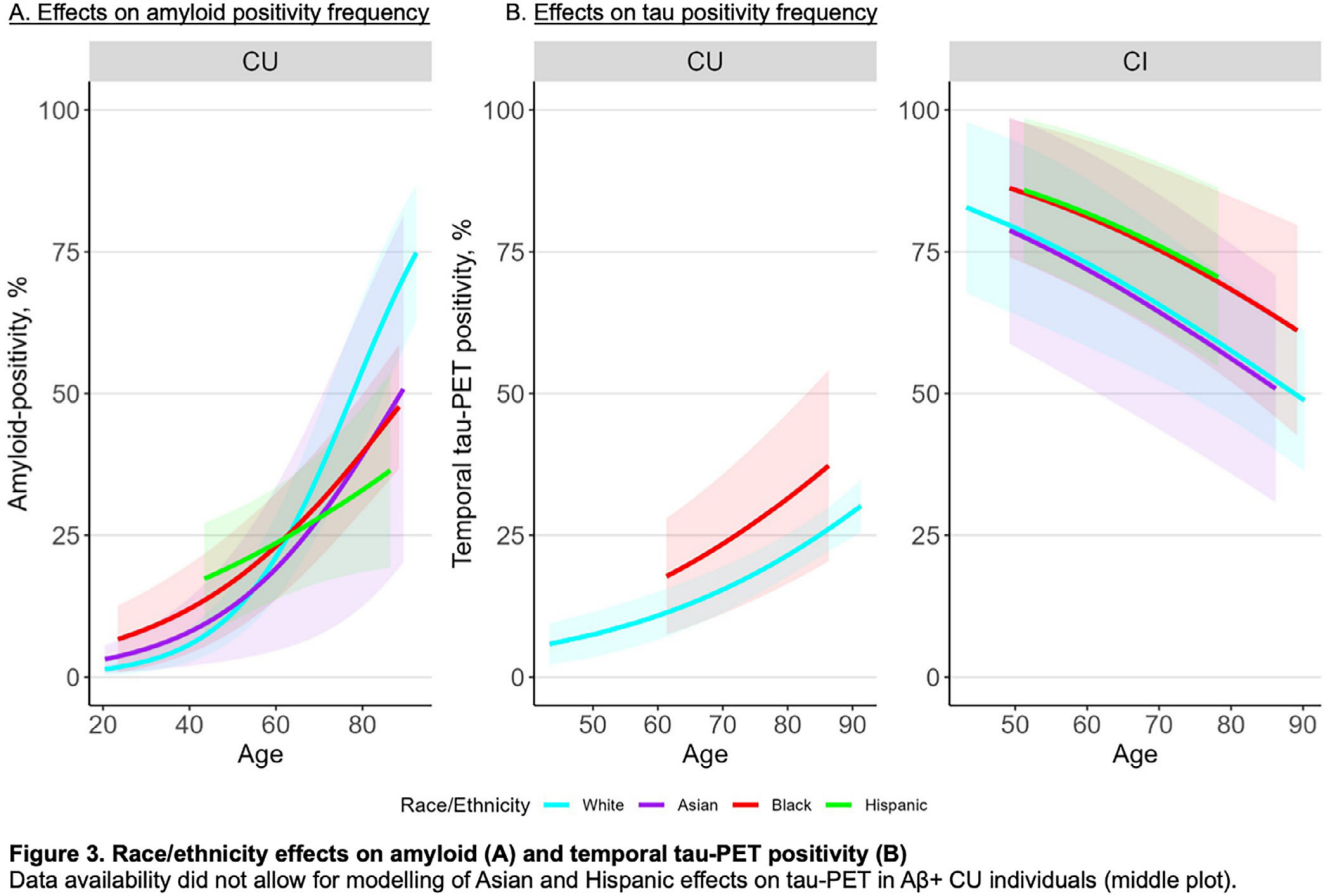# Sex, education and race/ethnicity have distinct effects on amyloid and tau‐PET status

**DOI:** 10.1002/alz70856_105387

**Published:** 2026-01-08

**Authors:** Colin Groot, Emma M. Coomans, Christopher C. Rowe, Vincent Dore, Azadeh Feizpour, Elsmarieke van de Giessen, Wiesje M. van der Flier, Yolande A.L. Pijnenburg, Pieter Jelle Visser, Anouk den Braber, Michael J. Pontecorvo, Samantha C. Burnham, Ian A. Kennedy, Ming Lu, William J. Jagust, Suzanne L. Baker, Theresa M. Harrison, Juan Domingo Gispert, Mahnaz Shekari, Carolina Minguillón, Ruben Smith, Niklas Mattsson‐Carlgren, Sebastian Palmqvist, Olof Strandberg, Erik Stomrud, Adam Brickman, José A Luchsinger, Jennifer J. Manly, Patrick J. Lao, William Charles Kreisl, Maura Malpetti, John T O'Brien, James B Rowe, Elena Jäger, Gérard N Bischof, Alexander Drzezga, Fang Xie, Xiaoxie Mao, Yihui Guan, Valentina Garibotto, Giovanni B Frisoni, Débora E. Peretti, Michael Schöll, Ingmar Skoog, Silke Kern, Reisa A. Sperling, Keith A. Johnson, Shannon Risacher, Andrew J. Saykin, Maria C. Carrillo, Brad Dickerson, Liana G. Apostolova, Gil D. Rabinovici, Henryk Barthel, Michael Rullmann, Konstantin Messerschmidt, Rik Vandenberghe, Koen Van Laere, Laure Spruyt, Ronald Petersen, Clifford R. Jack, Nicolai Franzmeier, Matthias Brendel, Johannes Gnörich, Tammie Benzinger, Julien Lagarde, Marie Sarazin, Michel Bottlaender, Sylvia Villeneuve, Judes Poirier, Sang Won Seo, Yuna Gu, Jun Pyo Kim, Beth Mormino, Christina B. Young, Hillary Vossler, Pedro Rosa‐Neto, Joseph Therriault, Nesrine Rahmouni, William Coath, David M Cash, Jonathan M Schott, Bernard J Hanseeuw, Yasmine Salman, Vincent Malotaux, Willemijn J. Jansen, Renaud La Joie, Howard J. Rosen, Sterling C Johnson, Bradley T Christian, Tobey J. Betthauser, Susan M. Landau, Sid E. O'Bryant, Oskar Hansson, Rik Ossenkoppele, ADNI, ADNI‐DOD, A‐Study, MCSA, Prevent‐AD

**Affiliations:** ^1^ Alzheimer Center Amsterdam, Neurology, Vrije Universiteit Amsterdam, Amsterdam UMC location VUmc, Amsterdam, Netherlands; ^2^ Department of Neurology, Alzheimer Center Amsterdam, Amsterdam Neuroscience, Vrije Universiteit Amsterdam, Amsterdam, Netherlands; ^3^ University of Melbourne, Melbourne, VIC, Australia; ^4^ Department of Nuclear Medicine and Centre for PET, Austin Health, Heidelberg, Vic, 3084, Australia, Heidelberg, VIC, Australia; ^5^ CSIRO, Brisbane, QLD, Australia; ^6^ Department of Molecular Imaging, Austin Health, Melbourne, VIC, Australia; ^7^ Department of Molecular Imaging & Therapy, Austin Health, Melbourne, VIC, Australia; ^8^ Vrije Universiteit Amsterdam, Amsterdam UMC location VUmc, Amsterdam, Netherlands; ^9^ Alzheimer Center Amsterdam, Neurology, Amsterdam UMC Location VUmc, Vrije Universiteit Amsterdam, Amsterdam, Netherlands; ^10^ Alzheimer Center Amsterdam, Department of Neurology, Amsterdam UMC, location VUmc, Amsterdam, Netherlands; ^11^ Eli Lilly and Company, Indianapolis, IN, USA; ^12^ Lawrence Berkeley National Laboratory, Berkeley, CA, USA; ^13^ University of California, Berkeley, Berkeley, CA, USA; ^14^ Barcelona?eta Brain Research Center (BBRC), Barcelona, Spain; ^15^ Barcelonaβeta Brain Research Center (BBRC), Pasqual Maragall Foundation, Barcelona, Spain; ^16^ Clinical Memory Research Unit, Lund University, Malmö, Skåne, Sweden; ^17^ Clinical Memory Research Unit, Department of Clinical Sciences Malmö, Lund University, Lund, Sweden; ^18^ Columbia University, New York, NY, USA; ^19^ Columbia University Irving Medical Center, New York, NY, USA; ^20^ Taub Institute for Research on Alzheimer's Disease and the Aging Brain, Vagelos College of Physicians and Surgeons, Columbia University, New York, NY, USA; ^21^ UK Dementia Research Institute at the University of Cambridge, Cambridge, United Kingdom; ^22^ University of Cambridge, Cambridge, Cambridgeshire, United Kingdom; ^23^ University of Cambridge, Cambridge, ‐, United Kingdom; ^24^ University of Cologne, Faculty of Medicine and University Hospital Cologne, Department of Nuclear Medicine, Cologne, Germany; ^25^ Huashan Hospital, Fudan University, Shanghai, Shanghai, China; ^26^ School of Medicine, Xiamen University, Xiamen, Fujian, China; ^27^ Department of Nuclear Medicine and PET Center, Huashan Hospital, Fudan University, Shanghai, China; ^28^ Faculty of Medicine, University of Geneva, Geneva, Geneva, Switzerland; ^29^ Geneva Memory Center, Geneva University Hospitals and University of Geneva, Geneva, Switzerland; ^30^ Laboratory of Neuroimaging and Innovative Molecular Tracers (NIMTlab), Geneva University Neurocenter and Faculty of Medicine, University of Geneva, Geneva, Switzerland; ^31^ Department of Psychiatry and Neurochemistry, University of Gothenburg, Mölndal, Västra Götalands län, Sweden; ^32^ Neuropsychiatric Epidemiology, Institute of Neuroscience and Physiology, Sahlgrenska Academy, Centre for Ageing and Health (AGECAP) at the University of Gothenburg, Gothenburg, Sweden; ^33^ Brigham and Women's Hospital, Harvard Medical School, Boston, MA, USA; ^34^ Massachusetts General Hospital, Boston, MA, USA; ^35^ School of Medicine, Indiana University, Indianapolis, IN, USA; ^36^ Indiana Alzheimer's Disease Research Center, Indiana University School of Medicine, Indianapolis, IN, USA; ^37^ Alzheimer's Association, Chicago, IL, USA; ^38^ Department of Neurology, Massachusetts General Hospital and Harvard Medical School, Boston, MA, USA; ^39^ Department of Radiology and Imaging Sciences, Center for Neuroimaging, Indiana University School of Medicine, Indianapolis, IN, USA; ^40^ Department of Neurology, University of California, San Francisco, San Francisco, CA, USA; ^41^ Department of Nuclear Medicine, University of Leipzig, Leipzig, Germany; ^42^ Leipzig University Medical Center, Leipzig, Germany; ^43^ Laboratory for Cognitive Neurology, Department of Neurosciences, KU Leuven, Leuven, ‐, Belgium; ^44^ KU Leuven and University Hospital Leuven, Leuven, Belgium; ^45^ Laboratory for Cognitive Neurology, KU Leuven, Leuven, Belgium; ^46^ Mayo Clinic, Rochester, MN, USA; ^47^ Institute for Stroke and Dementia Research (ISD), University Hospital, LMU Munich, Munich, Bavaria, Germany; ^48^ LMU University Hospital, Munich, Germany; ^49^ University Hospital, LMU Munich, Munich, Germany; ^50^ Washington University School of Medicine, St. Louis, WI, USA; ^51^ Université Paris‐Saclay, CEA, CNRS, Inserm, SHFJ, BioMaps, Orsay, France; ^52^ Neurologie de la Mémoire et du Langage, Université Paris Descartes, Sorbonne Paris Cité, INSERM UMR S894, Centre Hospitalier Sainte Anne, Paris, France; ^53^ StoP‐AD Centre, Douglas Mental Health Institute Research Centre, Montreal, QC, Canada; ^54^ Centre for Studies on Prevention of Alzheimer's Disease (StoP‐AD Centre), Douglas Mental Health University Institute, Montréal, QC, Canada; ^55^ Samsung Medical Center, Gangnam‐gu, Seoul, Korea, Republic of (South); ^56^ Samsung Medical Center, Sungkyunkwan University School of Medicine, Gangnam‐gu, Seoul, Korea, Republic of (South); ^57^ Department of Neurology and Neurological Sciences, Stanford University School of Medicine, Stanford, CA, USA; ^58^ Stanford University School of Medicine, Stanford, CA, USA; ^59^ McConnell Brain Imaging Centre, Montreal Neurological Institute, McGill University, Montreal, QC, Canada; ^60^ McGill University, Montreal, QC, Canada; ^61^ Montreal Neurological Institute, Montreal, QC, Canada; ^62^ Dementia Research Centre, UCL Queen Square Institute of Neurology, University College London, London, United Kingdom; ^63^ University College London, London, England, United Kingdom; ^64^ Institute of Neuroscience, UCLouvain, Brussels, Belgium; ^65^ Alzheimer Center Limburg, School for Mental Health and Neuroscience, Maastricht University, Maastricht, Limburg, Netherlands; ^66^ Memory and Aging Center, UCSF Weill Institute for Neurosciences, University of California, San Francisco, San Francisco, CA, USA; ^67^ University of California, San Francisco, San Francisco, CA, USA; ^68^ Wisconsin Alzheimer's Institute, University of Wisconsin School of Medicine and Public Health, Madison, WI, USA; ^69^ Department of Medical Physics, University of Wisconsin, Madison, WI, USA; ^70^ Wisconsin Alzheimer's Institute, University of Wisconsin‐Madison School of Medicine and Public Health, Madison, WI, USA; ^71^ Neuroscience Department, University of California, Berkeley, Berkeley, CA, USA; ^72^ University of North Texas Health Science Center, Fort Worth, TX, USA; ^73^ Amsterdam University Medical Center, Amsterdam, Netherlands; ^74^ Clinical Memory Research Unit, Department of Clinical Sciences Malmö, Faculty of Medicine, Lund University, Lund, Sweden

## Abstract

**Background:**

Sex, education and race/ethnicity are all associated with risk of Alzheimer's disease dementia. Here, we assess the effects of self‐reported sex, educational attainment and race/ethnicity on amyloid‐positivity, and tau‐PET‐positivity in 12,048 (7,394 cognitively unimpaired [CU], 2,177 MCI, and 2,477 dementia) individuals from 42 cohorts worldwide.

**Method:**

Logistic generalized estimating equations were used to estimate frequency of amyloid‐positivity (using cohort‐specific thresholds for amyloid‐PET [84%] or CSF) and tau‐PET‐positivity (cohort‐specific thresholds of 2SD above mean temporal uptake in amyloid‐negative controls). We assessed: i) sex and *APOEε4* (*N* = 10,098) associations, to complement earlier findings of a higher frequency of tau‐positivity in females, ii) effects of lower/higher education (*N* = 10,970; cohort‐specific median‐split), and iii) effects of race/ethnicity (non‐Hispanic White [hereafter: White], *N* = 4880; Asian, *N* = 116; Black or African‐American [hereafter: Black], *N* = 353; Hispanic, *N* = 356, only from Northern‐American cohorts). Outcomes were frequency of amyloid‐positivity in CU individuals only, and tau‐PET‐positivity in both amyloid‐positive (AB+) CU and cognitively impaired (CI, i.e. MCI and dementia) individuals. Interaction effects on the relationship between age and amyloid/tau‐positivity were assessed and only retained in the models when significant.

**Result:**

Female sex was associated with an *APOEε4*‐independent increased frequency of amyloid‐positivity (β=0.51[0.22], *p* = 0.02) in CU and increase of tau‐positivity in both AB+CU (β=0.27[0.08]) and AB+CI (β=0.37[0.08], both *p* <0.01). Remarkably, tau‐positivity frequencies of female *APOEε4* non‐carriers were equivalent to male *APOEε4* carriers in AB+CI (Figure 1). No significant sex*APOE interactions were observed.

In CU, higher education was associated with lower amyloid‐positivity frequency (β=‐0.12[0.05], *p* = 0.02). In contrast, among AB+CU, there was an age*education interaction effect that indicated more pronounced age effects on tau‐positivity in individuals with higher education (age*education:β_interaction_=0.03[0.01], *p* <0.01). There were no education effects in AB+CI (Figure 2).

In CU, an age*race/ethnicity interaction effect was observed across all non‐White groups compared to White (Hispanic:β_interaction_=‐0.05[0.01], *p* <0.01; Black:‐0.04[0.01], *p* <0.01; Asian:‐0.02[0.01], *p* = 0.04). This suggests that the impact of age on amyloid‐positivity was less pronounced in non‐White groups. Furthermore, in AB+CI, Hispanic ethnicity was related to higher tau‐positivity frequency than White (β=0.51[0.22], *p* = 0.02; Figure 3).

**Conclusion:**

In this multi‐center initiative comprised of clinical and community‐based cohorts, we observed that self‐reported sex, educational attainment and race/ethnicity were related to positivity‐frequencies of Alzheimer's disease pathology.